# Conservative Treatment of Bilateral Impacted Mandibular Canines Traction

**DOI:** 10.1155/2023/6943221

**Published:** 2023-08-01

**Authors:** Gustavo Armando Ruíz-Mora, Luis Ernesto Arriola-Guillén, Aron Aliaga-Del Castillo, Yalil Augusto Rodríguez-Cárdenas, Vinicius Dutra, Mabel Mejía-Milian

**Affiliations:** ^1^Division of Orthodontics, Faculty of Dentistry, Universidad Nacional de Colombia, Bogota, Colombia; ^2^Division of Oral and Maxillofacial Radiology, School of Dentistry, Universidad Científica del Sur, Lima, Peru; ^3^Division of Orthodontics and Division of Oral and Maxillofacial Radiology, School of Dentistry, Universidad Científica del Sur, Lima, Peru; ^4^Department of Orthodontics and Pediatric Dentistry, School of Dentistry, University of Michigan, Ann Arbor, MI, USA; ^5^Division of Oral and Maxillofacial Radiology, School of Dentistry, Universidad Nacional de Colombia, Bogota, Colombia; ^6^Department of Oral Pathology, Medicine, and Radiology, School of Dentistry, Indiana University, Indianapolis, Indiana, USA

## Abstract

The objectives of the treatment of impacted canines differ according to the characteristics of dental malocclusion. Traction of the tooth is a conservative and viable alternative, which allows for maintaining stability and occlusal function. The following case report describes the treatment of an 11.6-year-old female patient, who presented bilateral impaction of mandibular canines in contact with the midline, mixed dentition in the inter-transitional period, class I angle malocclusion, with light crowding teeth. The treatment comprised three phases. The first phase, verticalization of the mandibular canines in mixed dentition, was performed to pull the impacted bilateral mandibular canines towards the dental arch to achieve their verticalization, maintaining the molar relationship, and the position of the upper and lower incisors. In the second phase, osteogenic rest was planned to relieve post-traction stress while awaiting the replacement of the mixed dentition. Finally, the third phase in permanent dentition was to align and level canines within the arch after extraction of the deciduous canines. For the viability of the permanent mandibular canines in the dental arch, orthosurgical traction was implemented, with a traction system with closed nickel–titanium coil springs with a transitory rigid dental-mucous-supported anchorage device, which allowed control and protection of the adjacent teeth and movements with helical forces of a controlled three-dimensional range. The results of the treatment were adequate, achieving consolidated molar and canine relationships, overjet, overbite, and optimal facial balance.

## 1. Introduction

Dental impaction in the oral cavity is one of the most defying challenges in clinical practice, with the severity increasing when teeth that are indispensable for function, esthetics, and stability in the occlusion are involved, as in the case of the canines. The eruptive development of the mandibular canines should be monitored from early mixed dentition, evaluating their relationship with the deciduous teeth. The trajectory of the mandibular canine germ indicates the possibility of impaction [1]. Evaluation with diagnostic imaging is an adequate tool for detecting early anomalies in the eruptive development, aiding in treatment planning, and avoiding displacement of the impacted mandibular canine (IMbC) [2, 3]. Nevertheless, this alteration is commonly an incidental finding identified in routine radiographic images or clinical manifestations suggesting this possible clinical situation [3, 4].

The incidence of impaction of the mandibular canine is low, with the bilateral presentation being even less frequent and it is more common in females [3–5]. It is related to etiological mechanical or genetic factors, such as abnormal resorption of the deciduous canine or interruption of the development of its eruptive process [2]. Eruptive disturbances may also be influenced by pathologic anomalies, severe space discrepancies, narrow arcs, and delayed eruption, increasing the risk of dental impaction [3, 4]. Many IMbCs are displaced to the midline, producing the transmigration phenomenon [5].

The most common treatment options for IMbC are surgical extraction with the closing of the edentulous space with the premolar as a substitute, maintaining the deciduous canines or substitution with an osteointegrated implant, or by auto-transplantation of the impacted tooth, or surgical exposition and traction to the dental arc [6–8].

Treatment planning for the traction of an impacted tooth must consider several factors, such as the severity of the malposition, treatment time, orthodontic biomechanics, periodontal stability, relationship with adjacent teeth, surgical techniques, adhesive, and traction mechanism, and clinical experience [6, 9, 10]. Several reports on skeletal and dental anchorage mechanisms have described traction attachments, including metallic chains, metallic ligatures, elastic chains, and nickel–titanium closed coil springs, which affect the control, friction, soft tissue health, time, and magnitude of traction force [11–14].

This case report describes a biomechanical alternative performed in three phases from mixed dentition to permanent dentition, in a female patient with mandibular canines that were bilaterally impacted in the midline and displaced towards the buccal side.

## 2. Case Presentation

### 2.1. Publication Consent

The patient's mother signed the consent for the publication of this case report, and the patient gave her informed assent.

### 2.2. Diagnosis and Etiology

**Figure 1:**
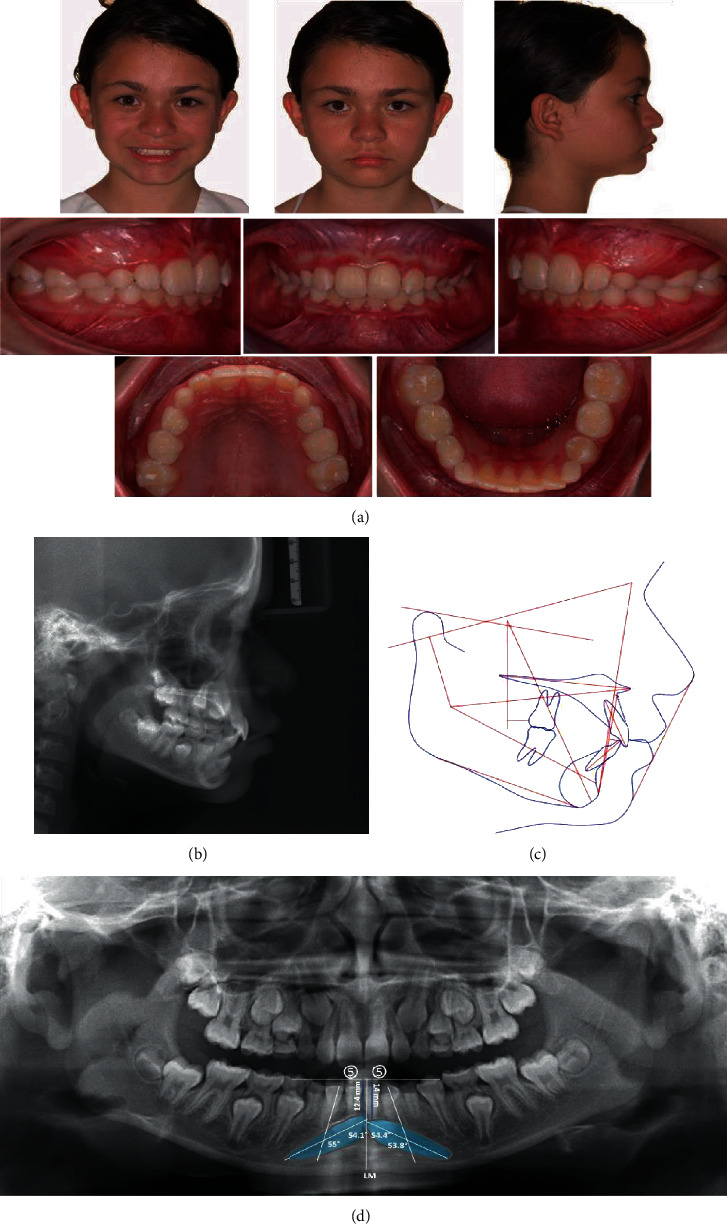
Initial clinical radiographic records. (a) Facial and intraoral photographs. (b) Lateral X-ray. (c) Cephalometric tracing. (d) Panoramic X-ray: right canine: impaction sector 5 (close to the midline, adapted from Ericson and Kurol [14]); impact level: middle root third; angle between the longitudinal axis of the canine about the midline: 54.1°; angle between the longitudinal axis of the canine and the longitudinal axis of the adjacent lateral: 55°; distance from the cusp of the canine to the occlusal plane: 12.4 mm. Left canine: impaction sector 5 (close to the midline); impact level: middle root third; angle between the longitudinal axis of the canine in relation to the midline: 55.4°; angle between the longitudinal axis of the canine and the longitudinal axis of the adjacent lateral 53.8°; distance from the cusp of the canine to the occlusal plane: 14 mm.

A female patient of 11 years and 6 months of age was referred for orthodontic consultation due to bilateral impaction of the mandibular canines. Clinical examination showed facial symmetry, a slightly convex profile, decreased lower facial height, labial competence, and a brachyfacial face type, without functional problems, and with no medical history of relevance. In the intraoral examination, mixed dentition was observed in the inter-transitional period, not coinciding with the chronological age due to a delay in the spontaneous eruption of the premolars and canines, although all the first permanent molar, upper and lower incisors, eight deciduous molars, and both upper and lower deciduous canines were present. The patient also presented a class I angle malocclusion, with no evidence of crowding and coinciding midlines (Figure 1).

In the orthopantomography dental germs of permanent premolars and molars in intraosseous evolution were visualized, and the bilateral ectopic position of the mandibular canines was detected, located meso-angulated, buccal, and in the midline, over the inferior third of the lower incisor roots. The permanency of the deciduous mandibular canines with no root resorption was confirmed (Figure 1). The severity of the impaction was diagnosed with anatomical mandibular landmarks, adapted from the study by Ericson and Kurol [14] on maxillary canines. In this case scenario, this report describes the condition of both buccally impacted canines located in the middle third of the incisor roots as measured in the panoramic radiograph. The longitudinal axis of the right canine with relation to the midline presented a 54.1° angle, and with relation to the longitudinal axis of the adjacent lateral incisor of 55°, with a distance from the occlusal plane of 12.4 mm; the left canine presented 55.4° with relation to the midline, 53.8° with the longitudinal axis of the adjacent lateral incisor, and a distance from the occlusal plane of 14 mm, located more buccally than the right canine (Figure 1).

The cephalometric measures indicated a skeletal class I (ANB = 1.1°), horizontal mandibular plane, diminished inferior facial height, upper incisors in adequate inclination, and pro-inclination of the lower incisors (Table 1).

Additionally, following the principles and recommendations of ALARA, cone-beam computed tomography (CBCT) images were taken to confirm the exact location of the impacted canines and their relationship with the lower incisors. The tomographic slices confirmed direct contact of the follicles and the incisor roots, without any evident damage, as well as the more buccal position of the left canine. The CBCT images were obtained with an i-CAT scanner (Imaging Sciences International, Hatfield, PA, USA) with the following configuration: 120 kV and 47.7 mA, with an exposure time of 20 seconds, a field of view of 8 cm × 8 cm, and a voxel size of 0.4 mm. The DICOM file was analyzed with the Dolphin 3D Imaging version 11.9 software (Chatsworth, CA, USA), allowing multiplanar and volumetric reconstruction.

### 2.3. Treatment Objectives

The objective of the treatment planning, in this case, was divided into three phases. The first phase involved the mixed dentition and was aimed at achieving the traction of both IMbCs into the arch to produce verticalization, whereas maintaining the molar relationship and incisor position. The second phase was planned as an osteogenic rest to alleviate the post-traction stress while awaiting the eruption of the permanent teeth. Finally, the objective of the third phase in the permanent dentition was to align and level both canines in the arch before the extraction of the deciduous canines, consolidating the molar and canine relationships, overjet, overbite, and an optimal facial balance.

### 2.4. Treatment Alternatives

Several treatment alternatives were considered for the resolution of this case report: (1) extraction of the IMbCs, preserving the deciduous canines; (2) extraction of the lower canines, the lower deciduous canines, and upper first bicuspids; (3) extraction of the lower permanent and deciduous canines, followed by the use of temporary anchorage devices to mesialize the lower posterior teeth; (4) extraction of lower permanent and deciduous canines, then retraction and uprighting of the lower incisor, and use of an orthopedic or functional appliance for stimulating forward growth potential; (5) auto-transplant of the impacted permanent canines; and (6) traction of permanent canines to the dental arch, considering other anchor resources and personal attachments in the traction of the canine.

The different treatment alternatives were presented to the patient's parents, and the final decision was to perform traction of the permanent mandibular canine into the dental arch. Then, a traction system was made of nickel–titanium closed spring coils with a rigid temporal mucodental supported device. This anchorage and traction system allowed control and protection of the adjacent teeth and movements with helical forces of a controlled three-dimensional (3D) range in the orthogonal axes *x*, *y*, and *z* [15].

### 2.5. Treatment Progress

The treatment progress was made up of three phases (Table 2). The first phase was in the mixed dentition, the main objective of which was achieving verticalization of both IMbCs.

**Figure 2:**
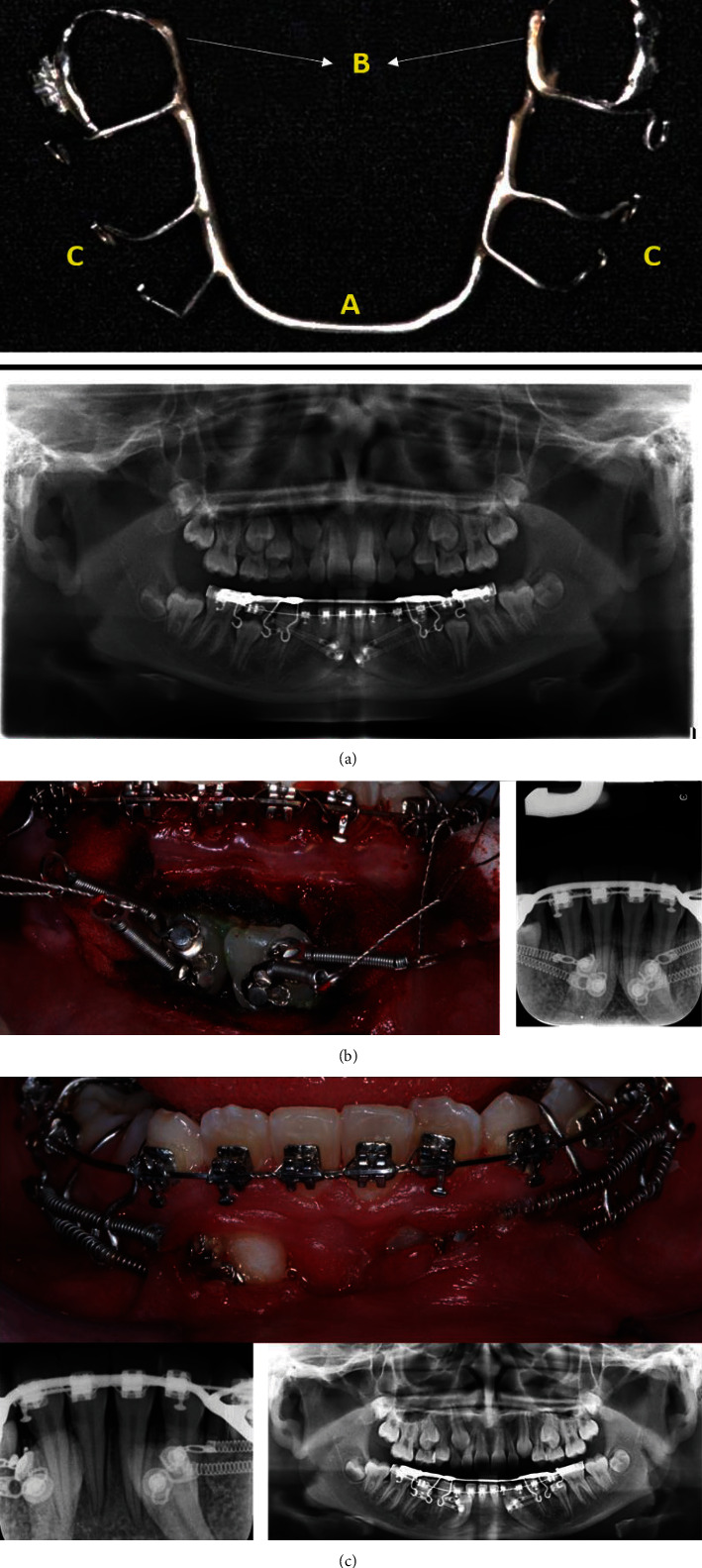
(a) Fixed device for temporary anchorage. (A) Lingual arch main support in 1.1 mm SS wire. (B) Prefabricated metal bands with lower first molar tubes. (C) Vertical hooks in 0.028″ SS wire, with bends towards the distal direction. (b) Trans-surgical radiographic and clinical records. (c) Three months of traction. Radiographic and clinical records.

Metallic brackets were placed on the mandibular teeth, slot 0.022^″^ × 0.028^″^ (Synergy; Rocky Mountain Orthodontics, Denver, CO, USA.), reaching a 0.016 × 0.022 CuNiTi arch-wire (Synergy; Rocky Mountain Orthodontics), including the deciduous molars. One month before surgical exposure of the canines performed with the closed technique, in which only the NiTi coil spring for traction was exposed, and before suturing, a temporary rigid anchorage device consisting of a lingual arch made with a 1.1 mm stainless steel (SS) arch-wire (Synergy; Rocky Mountain Orthodontics) was cemented in the first permanent molars. This was adapted to the lingual surface of the mandibular teeth, with proximal occluso-buccal extensions (SS 0.028″, Dentaurum, Ispringen, Germany) from canine to molar with the hooks in the distal direction on both sides of the dental arch (Figure 2).

The surgical intervention of the closed traction technique involved: (1) incision and flap lift, (2) osteotomy and exposure of dental follicles, (3) elimination of the pericoronocary follicles and complete clearance of the permanent canine clinical crown, without exceeding the cementoenamel junction, (4) isolation and blood control with hemostatic agents and drying, to ensure adhesion of the attachments, (5) placing the two nickel–titanium closed coil spring (Dentos, Daegu, South Korea) of 0.010 × 0.036 in diameter and 13 mm in length in each impacted canine, respectively, due to the distance exceeded in the activation, and the high resistance of the impacted tooth, and the relation of the coil springs with the anatomical structures and the surrounding dental landmarks. Each coil spring was activated immediately after suturing, with distal movement in the *x*-axis, vestibular movement in the *z*-axis, and extrusion in the *y*-axis. The activation was made every 6 weeks, extending the coil springs 13 mm, until 60% of their length, and by the gradual movement of each canine. Approximately 100 g of force was used to achieve traction of the tooth (Figure 2) [15].

**Figure 3:**
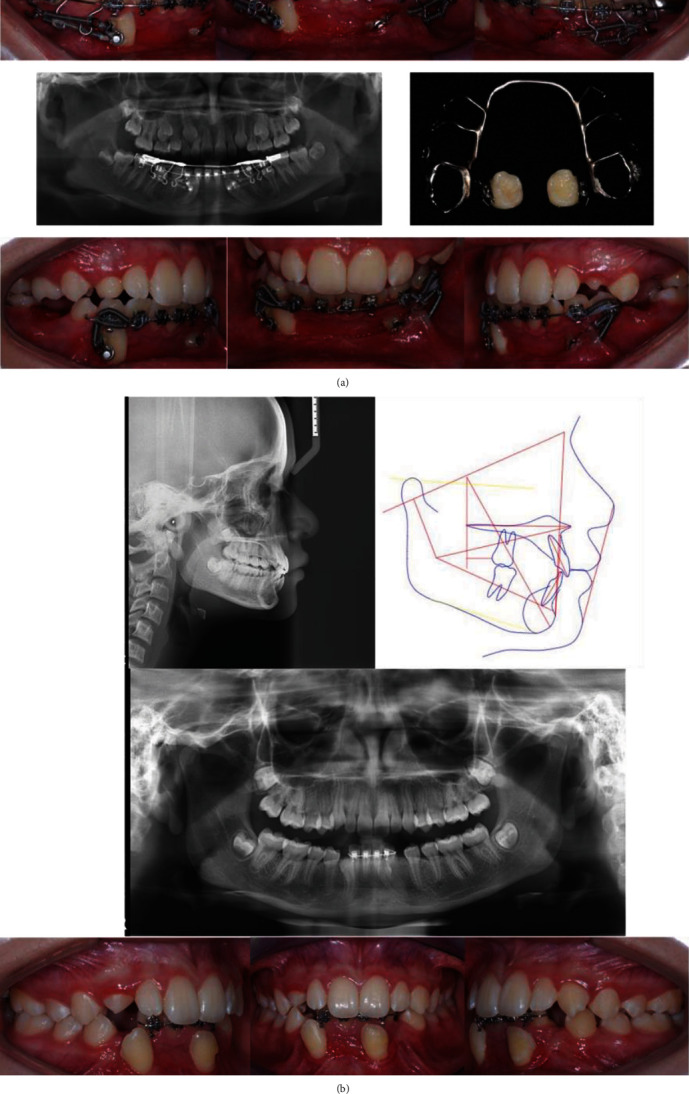
(a) Images of the first phase. Radiographic and clinical records. (b) Records of the start of the third phase of treatment.

Five months after the initial bracket placement, when the permanent canines were in a good vertical position parallel to the deciduous canines (Figure 3), the temporary anchorage device was removed, and the second treatment phase or osteogenic rest was started to relieve stress from the surrounding bone tissue, whereas maintaining the deciduous canine in place (Figure 3).

**Figure 4:**
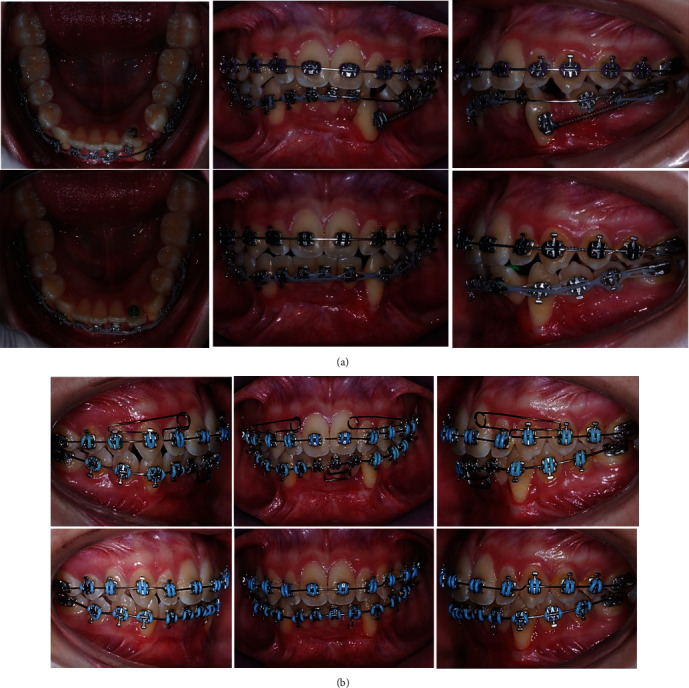
(a) Six months after the start of the third phase. (b) One year after initiating the third phase.

The third and final phase of the treatment started 18 months after the first visit, with a collection of clinical and radiography data (Figure 3). In this stage, the patient presented all permanent dentition, and the bracket placement was completed in both dental arches. At this time, the extraction of the deciduous canine was performed to start the immediate alignment of the permanent canines over the alveolus bone post-extraction. The alignment and leveling started using 0.016^″^ × 0.022^″^ CuNiTi arch-wire in both dental arches (Synergy; Rocky Mountain Orthodontics), followed by a 0.017^″^ × 0.25^″^ NiTi arch-wire, and finishing with 0.019^″^ × 0.025^″^ of SS (Synergy; Rocky Mountain Orthodontics). Additionally, an Australian 0.016″ arch-wire for anterior extrusion in the upper arch was used (Figure 4).

Due to the evident buccal malposition of the canines, there was a cortical reduction. To measure this, tomographic imaging was carried out with the consent of the patient's parents. Based on the transaxial slices and to move the canines in a lingual direction, set bends were made to incorporate the canine roots into the alveolar process (Figure 4).

### 2.6. Treatment Outcome Assessment

**Figure 5:**
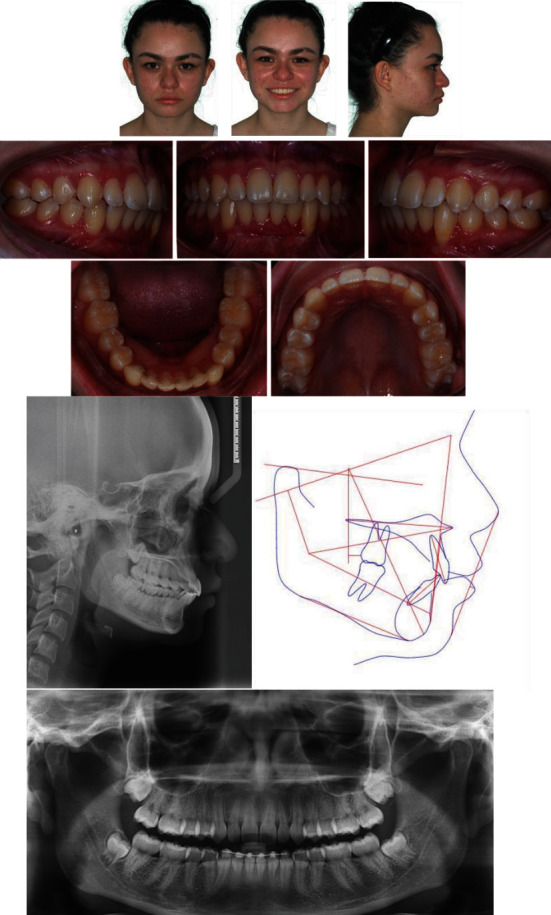
Extra-oral and intra-oral photographs and final radiographic-cephalometric records.

The patient presented good dental alignment, molar class I with adequate lateral and anterior functional guides. The clinical records showed adequate dental arch form, good inter-arch engagement, ideal overjet and overbite, and coinciding midlines, as well as an acceptable facial profile with few variations (Figure 5).

**Figure 6:**
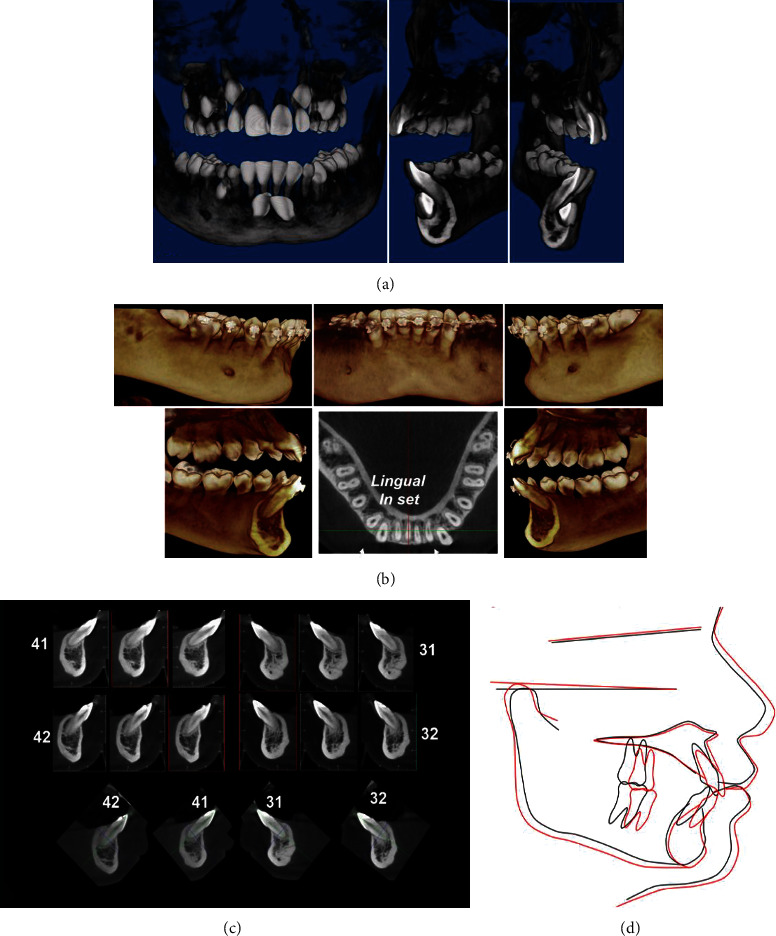
(a) Pre-treatment tomographic images. (b) Intermediate tomographic images showing the absence of buccal cortical at the level of the mandibular canines. (c) Post-treatment transaxial tomographic slices of lower incisors. (d) Superimposition of initial and final phase tracings.

The post-treatment panoramic radiograph showed dental alignment, conservation of the integrity of the anterior teeth, and the mandibular canine maintained a good root length. In the cephalometric examination, the mandibular incisor inclination had a similar position, whereas the inclination of the upper incisors had moderately increased (Table 1; Figure 6).

No periodontal disease was observed, with the periodontal probe showing good adherence to the gum (Hu-Friedy). Furthermore, a beneficial increased crown length was observed, with moderate cervical exposure of the crown of both canines. A lower lingual fixed retainer was adapted from canine to canine (Figure 5).

## 3. Discussion

The risk of teeth impaction is greater in patients with late eruption, being more common in females [5, 16]. In this clinical case, a female patient with late mixed dentition was treated. The diagnostic severity of the mandibular canine impaction was determined using anatomical parameters, and references adapted from the study of Ericson and Kurol on maxillary canines [14, 17]. The final diagnosis was an ectopic mesioangulated buccal eruption of both permanent mandibular canines, over the midline and in follicular contact with the roots of the lower central incisor. The complexity and severity of this clinical case led to the design of a biomechanical traction and anchorage system, which has not previously been described for IMbCs.

Before and after the traction treatment, 3D images were used for the diagnosis, treatment, and follow-up using different axial, coronal, and sagittal slices, allowing evaluation of the severity of the malposition, location of the ectopic tooth related to the alveolar cortical, the repercussion on the neighboring tooth, and possible bone losses [18]. The follow-up CBCTs were used to reevaluate the biomechanics of the lingual movement of the roots of the canines inside the bone by in-set bends in the first order and required torque movements.

Different treatment alternatives for IMbCs have been reported [19]. The most common alternative is surgical extraction of the impacted canine [17, 20–24]. Other alternatives are surgical exposure treatments and orthodontic traction [6, 7, 25]. In the present case, some treatment alternatives were considered, mainly extraction of the IMbCs and preservation of the deciduous canines, or the extraction of the lower canines, the lower deciduous canines, and replacement of these teeth with bicuspids and advancing the lower posterior sector. However, the possibility of producing a canine guide and the consequent esthetics prevailed over the patient's choice of traction for the affected teeth. Therefore, the treatment of this case was planned in three phases. In the first phase involving mixed dentition in the first transitional period, traction and verticalization of the IMbCs were performed with a reinforced anchoring device until a parallel position was achieved with the deciduous canines in the arch. In the second phase in the mixed dentition second transitional period, osteogenic rest and extraction of deciduous canines were performed, and finally, third phase involved alignment and leveling of the permanent canines within the alveolar bone with complete orthodontic arches. The extraction of the deciduous canines was postponed until the end of the second phase and the beginning of third phase to take advantage of the osteoid-osteopenic period of the fresh deciduous alveolus in the healing processes.

The effectiveness and need for the use of anchorage resources for the traction of impacted teeth are important. Compared with a temporary anchorage device-type skeletal anchorage, a customized rigid anchor cemented with bands was preferred [11, 26]. Tooth-supported anchorage devices are a viable alternative for controlled traction of bilaterally IMbCs, as they are low-cost, transitory devices that provide protection to the teeth and do not involve tissues compared with the adverse effects of action and reaction forces, thus avoiding important alterations in the patient's occlusion and the immersion of the springs in the mucoperiosteum [6, 27, 28]. Additionally, as the treatment has been completed without the use of mini-screws, the risk of root injury was avoided [29–31]. In this treatment alternative, a customized dentomucosal anchorage device with buccal extensions was adopted during the first phase. The anchoring system is efficient when combined with the nickel–titanium closed coil springs, which allow adequate force management and continuous preservation of the periodontal tissues throughout the traction process. Furthermore, they are not susceptible to deformation or fatigue, facilitating the implementation of traction with helical forces controlled in magnitude, direction, and direction, with distribution towards the distal direction in the *x*-axis, vestibular in the *y*-axis, and extrusive in the *z*-axis, providing superelasticity, memory, and a wide range of activation with light continuous forces, in a total traction period of approximately 6 months [12, 28, 32–35].

Although lower incisor resorption due to canine impaction is infrequent [36–38], sequelae due to root resorption have been described in the upper maxilla, but are infrequent in the mandible [15], favoring the selection of orthosurgical traction treatments, maintaining the permanent canines in the dental arch. The 3D imaging showed no root involvement of the incisors despite the severity of impaction and the bilateral frictional movement of the canine follicles on the root cement of the incisors (Figure 6). Another challenge was periodontal health, which can be affected by various factors, such as the type of surgical procedure selected to expose the crown of the impacted tooth, the eruption of the teeth through the attached gingiva or mucosa, the hygiene methods used, and the anatomical characteristics of the mandibular bone, including reduced bicortical width and alveolar rigidity [39]. It has been shown that surgical procedures closed by the buccal approach, such as that used in this case report, offer better results [40–44]. Nevertheless, coronal elongation of the canines was observed in the patient, with cervical exposure and possible projection of the roots through the attached gingiva margin due to the narrowness of the alveolar bone corridor, for which it was recommended to keep the canine and anteroinferior area in optimal hygiene conditions and monitor the stability of the occlusion.

The cephalometric findings showed proinclination of the upper and lower incisors due to treatment without extraction and the location of the canine impacted in the dental arch, producing an evident increase in the length, perimeter, and width in both arches. Another reason for the proinclination could be the need for canine and anterior functional guides [15, 35].

The length of treatment was prolonged due to predetermination of the chronological and dental age, the conservatory biomechanics of verticalization of the canines contacting the incisors, and the three-phase treatment plan with long resting periods for achieving morphologic and functional occlusal stability.

The case report was completed with good posterior occlusion settlement following the three-phase treatment, demonstrating that with light forces acceptable occlusion can be achieved. However, a limitation of this intervention is the great biological variability of the patients, which may not allow the same good results and may lead to the development of unexpected side effects. Therefore, more studies with larger sample sizes are needed to establish the most adequate procedure to perform in patients with impaction of mandibular canines.

## 4. Conclusion

The presence of bilaterally IMbCs is a diagnostic and therapeutic challenge for orthodontists. Nevertheless, with a good tridimensional plan, the use of biological forces and the implementation of different anchorage and control traction biomechanical methods can achieve optimum results, constituting a viable treatment alternative for orthodontists.

## Figures and Tables

**Table 1:** Cephalometric values.

Measurements	NORM	SD	Pre-treatment	Started the second phase	Final
SNA (°)	82	3.5	88	84.2	84
SNB (°)	80	3	86.9	82.2	82.7
ANB (°)	2°	2.4	1.1	2	2.3
OP-SN (°)	14.4	2.5	11.4	11.4	9.2
SN-GoGn (°)	32.9	5.2	20.1	20.6	18.6
FMA (°)	23.9	4.5	19.8	14.5	11.4
ENA-ME (mm)	66.7	4.1	54.01	52.10	53.20
U1-L1 (°)	130	6.0	118.4	112.7	109.8
U1-PP (°)	110	5	109.2	113.4	116.8
U1.NA (°), U1-NA (mm)	22.8, 4.3	5.7, 2.7	19.4, 4	26.5, 4.9	28.2, 5.9
L1.NB (°), L1-NB (mm)	25.3, 4.0	6.0, 1.8	42.5, 2.6	38.8, 4.4	39.8, 6.4
IMPA (L1-MP) (°)	95	7.0	112.4	113.8	115.8
Overjet (mm)	2.5	2.5	3.7	3.1	2.3
Overbite (mm)	2.5	2.0	4.8	3.1	1.5
Upper lip to E-plane (mm)	0	2.0	1.73	0.44	−0.90
Lower lip to E-plane (mm)	0	2.0	2.32	−0.91	−0.69
Nasolabial angle (°)	95	5.0	88.90	75.08	67.00

**Table 2:** Three phases of the therapy for the case report.

Phase I: active traction	Phase II: osteogenic rest	Phase III: full fixed orthodontic appliances
Goal: Upright and traction of both impacted mandibular canines.	Goals: Relieve stress from the neighboring tissues.	Goal: Comprehensive orthodontic treatment.
Alignment and leveling: Metallic brackets (0.022^″^ × 0.028^″^ slot) in the mandibular arch including the primary teeth present. CuNiTi 0.016^″^ × 0.022^″^ arch-wire (1 month before surgical exposure).	Maintain permanent canines upright and parallel to primary canines.	Bonding full fixed orthodontic appliance: Include all permanent teeth. Metallic brackets (0.022^″^ × 0.028^″^ slot).
Temporary rigid anchorage device: Lower lingual holding arch (SS, 1.1 mm) associated with interproximal occluso-buccal extensions (SS .028″) with hooks in the distal direction.	Removal of the temporary anchorage device.	Extraction of the lower primary canines and immediate retraction, leveling, and alignment of the permanent canines.
Surgical exposure: Incision, flap, osteotomy, removal of dental follicles, clearance of canine crown, bonding of attachments, and NiTi closed coil springs (0.010 mm × 0.036 mm diameter and 13 mm long).	Segmented arch to maintain coil springs tied.	Arch-wire sequence: Lower: CuNiTi 0.016^″^ × 0.022^″^, NiTi 0.017^″^ × 0.025,^″^ SS 0.019^″^ × 0:025^″^, SS 0.021^″^ × 0.025^″^. Australian .016^″^ for anterior extrusion and finishing.
Activation: Each 6 weeks. Traction force: 100 g.	Monitor root development of permanent teeth.	Upper: CuNiTi 0.016^″^ × 0.022^″^, NiTi 0.017^″^ × 0.025^″^, SS 0.016^″^ × 0.22^″^ (with boot loop for incisor derotation), SS 0.019^″^ × 0.025^″^, SS 0.021^″^ × 0.025^″^.
Phase I time: 5 months.	Phase II time: 13 months.	Phase III time: 18 months.

## Data Availability

The intra- and extra-oral figures and cephalometric data used to support the findings of this study are included within the article. For any information required, contact the corresponding author or Dr. Gustavo Ruíz responsible for managing the clinical case. garruiz@gmail.com, Private Dental Clinic luchoarriola@gmail.com.
